# A Universal *Mariner* Transposon System for Forward Genetic Studies in the Genus *Clostridium*

**DOI:** 10.1371/journal.pone.0122411

**Published:** 2015-04-02

**Authors:** Ying Zhang, Alexander Grosse-Honebrink, Nigel P. Minton

**Affiliations:** Clostridia Research Group, BBSRC/EPSRC Synthetic Biology Research Centre (SBRC), School of Life Sciences, University of Nottingham, Nottingham, United Kingdom; Universidad Andres Bello, CHILE

## Abstract

DNA transposons represent an essential tool in the armoury of the molecular microbiologist. We previously developed a *catP*-based mini transposon system for *Clostridium difficile* in which the expression of the transposase gene was dependent on a sigma factor unique to *C*. *difficile*, TcdR. Here we have shown that the host range of the transposon is easily extended through the rapid chromosomal insertion of the *tcdR *gene at the *pyrE *locus of the intended clostridial target using Allele-Coupled Exchange (ACE). To increase the effectiveness of the system, a novel replicon conditional for plasmid maintenance was developed, which no longer supports the effective retention of the transposon delivery vehicle in the presence of the inducer isopropyl β-D-1-thiogalactopyranoside (IPTG). As a consequence, those thiamphenicol resistant colonies that arise in clostridial recipients, following plating on agar medium supplemented with IPTG, are almost exclusively due to insertion of the mini transposon into the genome. The system has been exemplified in both *Clostridium acetobutylicum *and *Clostridium sporogenes*, where transposon insertion has been shown to be entirely random. Moreover, appropriate screening of both libraries resulted in the isolation of auxotrophic mutants as well as cells deficient in spore formation/germination. This strategy is capable of being implemented in any *Clostridium* species.

## Introduction

*Clostridium* is an ancient genus that comprises a large number of Gram-positive, rod-shaped bacteria. Obligate anaerobes, they are capable of producing endospores and are both of medical and industrial importance. The more notorious members of the genus are responsible for devastating infectious diseases (*Clostridium difficile*) and intoxications (*Clostridium botulinum*, *Clostridium perfringe*ns, and *Clostridium tetani*) of humans and animals [1,2]. The majority of its members are, however, non-pathogenic and are being investigated as the chassis for the sustainable production of chemicals and fuels, most notably solventogenic species, such as *Clostridium acetobutylicum*, *Clostridium beijerinckii*, *Clostridium thermocellum* and *Clostridium autoethanogenum* [3,4]. Yet other species, most notably *Clostridium sporogenes* and *Clostridium novyi*, have potential medical applications as the delivery vehicle of novel anti-tumour agents [5,6].

The growing importance of the genus has in recent times stimulated the development of genetic tools which may be deployed to both better understand clostridial biology and implement key process improvements. Accordingly, substantive progress has been made in the derivation of methods for directed gene knock-out and knock-in, including group II intron retargeting methods [7,8] as well as various procedures for undertaking allelic exchange using recombination-based strategies [9–14]. These methods may be deployed in classical reverse genetic approaches. Some progress has also been made in devising tools that can be used in forward genetics, where random mutants with the desired phenotype are isolated, and the nature of the genotype responsible is determined. The most useful tool in this context is a transposon. In this respect, *mariner*-based transposon mutagenesis systems offer the greatest utility [15,16], having been used in many different bacterial species, including low-GC-content Gram-positive species [17–21]. They insert into a ‘TA’ target site through a “cut-and-paste” mechanism [22], which makes them an ideal mutagen for clostridia.

To date, the successful use of a *mariner-*based mutagenesis system has been reported in *C*. *difficile* [15] and *C*. *perfringens* [16]. Both employed a similar plasmid-based strategy, in which the transposase gene was placed under the control of a conditional promoter which mitigated against expression of the transposase in the *E*. *coli* donor. This strategy ensures stability of the element in *E*. *coli*, both during the construction of the plasmid and prior to its transfer to the clostridial recipient. In both cases, the transposase was supplied in trans, and mediated the transfer of a mini-transposon element in a *catP* gene encoding resistance to chloramphenicol (Cm) / thiamphenicol (Tm), flanked by the two ITRs.

In the system deployed in *C*. *difficile* R20291, expression of the *Himar1 C9* transposase gene was under the control of the promoter of the *C*. *difficile* toxin B gene, *tcdB*. This promoter (P_tcdB_) is exclusively recognised by a specialised class of sigma factor, TcdR, which belongs to a family that is unique to toxinogenic clostridial species [15]. The utility of the system for forward genetic studies was demonstrated through the isolation of mutants defective in germination, (*cspBA*), and uracil metabolism, (*pyrB*). One drawback of the method was that whilst the delivery vehicle employed was segregationally unstable pseudo-suicide vector, it required a minimum of two passages of the recipient bacteria to eradicate the *Himar1 C9* transposase encoding plasmid. This limits the systems utility in high-throughput mutagenesis strategies. In the case of the system developed for *C*. *perfringens* strain 13, a counter-selection marker (the *galK* gene) was incorporated into the backbone of the transposon delivery vector and used in a host (strain HN13) in which the *galK* (galactokinase) and *galT* (Gal-1-phosphate uridylyltransferase) genes were deleted [16]. In the presence of 3% galactose, cells carrying a functional *galK* gene are unable to grow. Thus, by plating cells carrying the delivery vehicle on Tm-containing agar media supplemented with galactose, only transposon insertions that have lost the plasmid are able to grow. The system was successfully used to undertake a large scale screen for mutants lacking in gliding motility on agar plates. Whilst this experiment demonstrated the utility of this approach, the requirement for specific mutants lacking functional *galK* and *galT* genes is not ideal.

In the present study we have improved the utility of the system through the derivation of a transposon delivery vehicle that is conditional for replication, providing the facility for rapid plasmid loss after transfer into the clostridial host. Moreover, we have devised a simple strategy whereby the conditional expression of the transposase in the target clostridial host can be effected through the genomic incorporation of the *tcdR* gene. The system has been exemplified in two different clostridial species, *C*. *acetobutylicum* and *C*. *sporogenes*, and is potentially universally applicable to many different *Clostridium* spices.

## Materials and Methods

### Bacterial strains and media

Bacterial strains utilised in this study are listed in Table 1. *E*. *coli* strains were grown in Luria-Bertani medium at 37°C. *Clostridium spp* were cultured under anaerobic condition in an anaerobic cabinet (MG1000 Anaerobic Work Station, Don Whitley Scientific Ltd) containing an atmosphere of 80% nitrogen, 10% hydrogen and 10% carbon dioxide. Antibiotics were used at the following concentrations: erythromycin (Em), 20 μg/ml, thiamphenicol (Tm), 15 μg/ml for *Clostridium spp*; Em, 500 μg/ml, chloramphenicol (Cm), 25 μg/ml, tetracycline (Tc), 10 μg/ml for *E*. *coli*.

**Table 1:** Bacterial strains and plasmids used in this study.

Strain or Plasmid	Relevant characteristics^a^	Source^b^
Bacterial Strains		
*Clostridium acetobutylicum* ATCC 824	Wild type	ATCC
*Clostridium sporogenes* NCIMB 10696	Wild type	NCIMB
*E*. *coli* TOP10		Invitrogen
CRG3011	*C*. *acetobutylicum* ATCC 824 with *tcdR* inserted at *pyrE* locus	This study
CRG3817	*C*. *sporogenes* NCIMB 10696 *with tcdR* inserted at *pyrE* locus	This study
Plasmids		
pMTL83251	*Clostridium* modular plasmid used for construction of IPTG inducible promoter system. Em^R^	[32]
pMTL-ME6C	ACE vector for DNA integration at *pyrE* locus in *C*. *acetobutylicum* 824. Cm^R^	[10]
pMTL-YZ29	ACE vector for DNA integration at *pyrE* locus in *C*. *sporogenes* NCIMB 10696. Cm^R^	This study
pMTL-ME6C-tcdR	ACE plasmid for *tcdR* integration at *pyrE* locus in *C*. *acetobutylicum* 824. Cm^R^	This study
pMTL-YZ29-tcdR	ACE plasmid for *tcdR* integration at *pyrE* locus in *C*. *sporogenes* NCIMB 10696. Cm^R^	This study
pMTL83251-lacI	*Clostridium* modular plasmid with conditional replicon	This study
pMTL83251-lacI-T	*Clostridium* modular plasmid with conditional replicon and a *fdx* terminator	This study
pMTL87250	*Clostridium* modular plasmid with conditional replicon	This study
pMTL-YZ13	Transposon plasmid with pCB102 replicon	This study
pMTL-YZ14	Transposon plasmid with conditional replicon	This study
pMTL82254-PtcdB	*Clostridium* modular plasmid with *catP* reporter expressed by promoter *tcdB*	This study
pMTL82254-Pfdx	*Clostridium* modular plasmid with *catP* reporter expressed by promoter *fdx*	This study

^a^ Cm^R^, chloramphenicol/thiamphenicol resistance gene; Em^R^, erythromycin resistance gene

^b^ ATCC, American Type Culture Collection; NCIMB, The UK's National Collection of Industrial, Food and Marine Bacteria

*C*. *acetobutylicum* ATCC 824 and its mutant derivatives were grown in Clostridial Growth Medium (CGM) [23] for routine manipulations, or Supplemented Clostridium Basal Medium (CBMS) for fermentation assays. CBMS was based on Clostridium Basal Medium (CBM) as previously described[24] with two modifications. Glucose was added at a final concentration of 50 g/l, and 5 g/l calcium carbonate was added as a buffering agent. P2 medium [25] with 20 g/l glucose was used to screen for auxotrophic mutants.

*C*. *sporogenes* NCIMB 10956 and its mutant derivatives were grown in TYG medium (2% trypticase, 0.5% peptone, 0.1% glucose, 0.5% yeast extract and 0.1% cysteine-HCl) [26] for routine manipulations, or P2Y medium for fermentation assays. P2Y medium is P2 medium with some modifications, in which glucose was added at a final concentration of 50 g/l, 5 g/l calcium carbonate was added as a buffering agent and 2 g/l yeast extract was added for extra amino acids. MACC medium [27] with 20 g/l glucose was used to screen for auxotrophic mutants.

### Plasmids, primers, DNA techniques

Plasmids and primers used in this study are listed in Tables 1 and 2, respectively. Chromosomal DNA preparation, plasmid isolation and purification of DNA fragments from agarose gels were carried out using the DNeasy Tissue kit, the QIAprep Miniprep kit and the QIAquick Gel Extraction kit, respectively (Qiagen, UK). Restriction enzymes were supplied by New England Biolabs and were used according to the manufacturer’s instructions. *E*. *coli* strains were transformed by electroporation using a Gene-Pulser (Bio-Rad), as recommended by the manufacturer. PCR amplifications were carried out using the KOD Hot Start Master Mix (Merck). Oligonucleotides used in this study are detailed in Table 2, which were synthesised by Eurofins MWG Operon, Germany.

**Table 2:** Oligonucleotide primers used in this study.

	Sequence (5’-3’)	Description
Del-traJ	AAAAAAGCTTATAATTATCCTTATTGGACTTTCAAGTGCGCCCAGATAGGGTG	Delete traJ in pMTL5401Fcat
Del-traJ-antisense	CAGATTGTACAAATGTGGTGATAACAGATAAGTCTTTCAATTTAACTTACCTTTCTTTGT	Delete traJ in pMTL5401Fcat
YZ4	TGAACGCAAGTTTCTAATTTCGATTTCCAATCGATAGAGGAAAGTGTCT	Amplify IPTG cassette
YZ35	GCTGATATGGTAATGAAGGG	Amplify IPTG cassette
NotI/AscI	AACAGCTATGACCGGCGCGCCGCTCACTGCCCGC	Change NotI site to AscI in pMTL83251-lacI
NotI/AscI-antisense	GCGGGCAGTGAGCGGCGCGCCGGTCATAGCTGTT	Change NotI site to AscI in pMTL83251-lacI
tcdR-F	GCTATATCAAGTGCTAAAGGTC	Amplify tcdR gene
tcdR-R	AAAAAAGCTTATAATTATCCTTAGCGGTCCAAGACGTGCGCCCAGATAGGGTG	Amplify tcdR gene
catP-INV-F1	CAGATTGTACAAATGTGGTGATAACAGATAAGTCCAAGACTCTAACTTACCTTTCTTTGT	Inverse PCR [15]
catP-INV-R1	TGAACGCAAGTTTCTAATTTCGATTACCGCTCGATAGAGGAAAGTGTCT	Inverse PCR[15]
catP-INV-R2	GGATCTGTAATAACCCATAAAG	Sequencing of inverse PCR products[15]

To establish whether transposition had occurred, inverse PCR (INV) was performed according to the procedure of Cartman and Minton. [15]. All DNA sequencing was carried out by Source BioScience, United Kingdom.

To identify the genomic location of transposon insertions, sequence data were analyzed using DNASTAR (www.dnastar.com) and compared to the genome sequences using Artemis (www.sanger.ac.uk/resources/software/artemis/). The sequences of *C*. *acetobutylicum* ATCC 824 genome and megaplasmid pSOL1 are published (Refseq number NC_003030.1 and NC_001988.2; GenBank accession number AE001437 and AE001438) [28]. The GenBank accession number of *C*. *sporogenes* NCIMB 10956 is CP009225.

### Plasmid transfer in *Clostridium* species

*C*. *acetobutylicum* and *C*. *sporogenes* were transformed as described previously [29]. For *C*. *acetobutylicum*, prior to transformation, plasmid DNA was purified from *E*. *coli* TOP10 cells containing plasmid pAN2. This plasmid contains the ϕ3TI methyltransferase gene of *B*. *subtilis* phage ϕ3tI, which protects DNA from *C*. *acetobutylicum* Cac824I DNA restriction activity [30].

### Construction of plasmids

**Fig 1:**
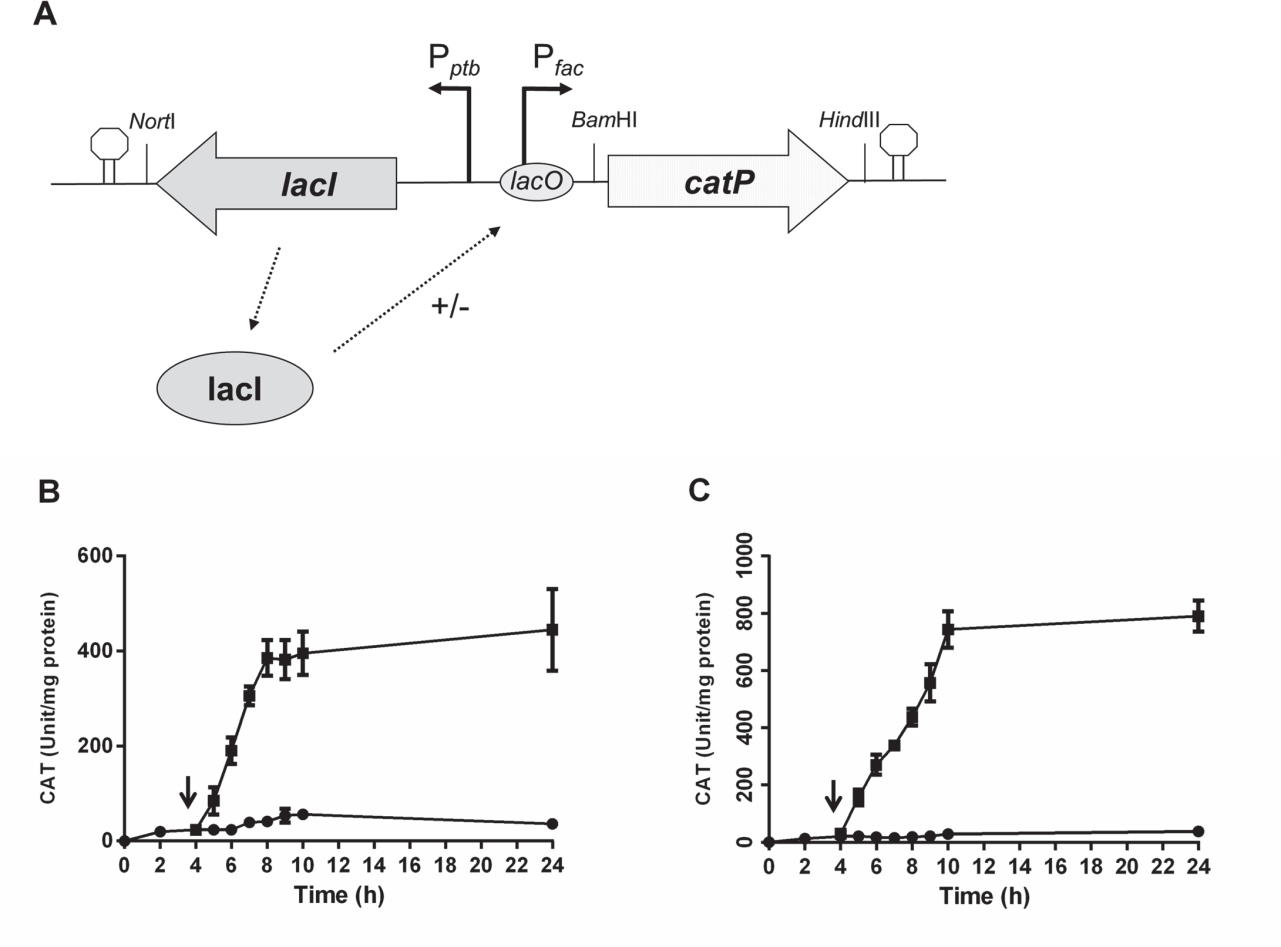
Schematic diagram of the *lac*-based, IPTG inducible expression cassette in pMTL-YZ2 (A), and the demonstration of its function in *C*. ***acetobutylicum* (B) and *C*. *sporogenes* (C). A**: Schematic diagram of the *lac*-based, IPTG inducible expression cassette. Key: LacI is the LacI repressor protein gene. LacI binds to the indicated *lacO* region, blocking transcription from the P_**fac**_ promoter. The P_**ptb**_ promoter (derived from the *C*. *beijerinckii* gene encoding phosphotransbutyrylase) directs the transcription of the *lacI* gene. **B** and **C**: IPTG induction of CAT production in of *C*. *acetobutylicum* (**B**) and *C*. *sporogenes* (**C**) harbouring pMTL-YZ2. Circles equate to cells which received no IPTG, squares represents samples from cells that were induced with IPTG. The arrow indicates the time of adding IPTG. Activity is expressed as units of CAT activity per mg of soluble protein.

Plasmid pMTL5401Fcat is a previously constructed plasmid [30] which mediates the IPTG inducible expression of the *catP* gene. To simplify and construct an IPTG inducible promoter cassette, a 982 bp DNA intervening region encompassing *oriT*/*traJ* was deleted from plasmid pMTL5401Fcat using the QuikChange Site-Directed Mutagenesis Kit (Stratagene) with primers Del-traJ and Del-traJ-antisense. Having deleted the *traJ* region, the 2728 bp *lac* inducible promoter cassette containing *lacI*, P_ptb_, P_fac_ and *cat* was then PCR amplified using primers YZ4 and YZ5 and cloned into pMTL83251 via NotI/HindIII, resulting in plasmid pMTL83251-YZ2 (Fig 1A).

**Fig 2:**
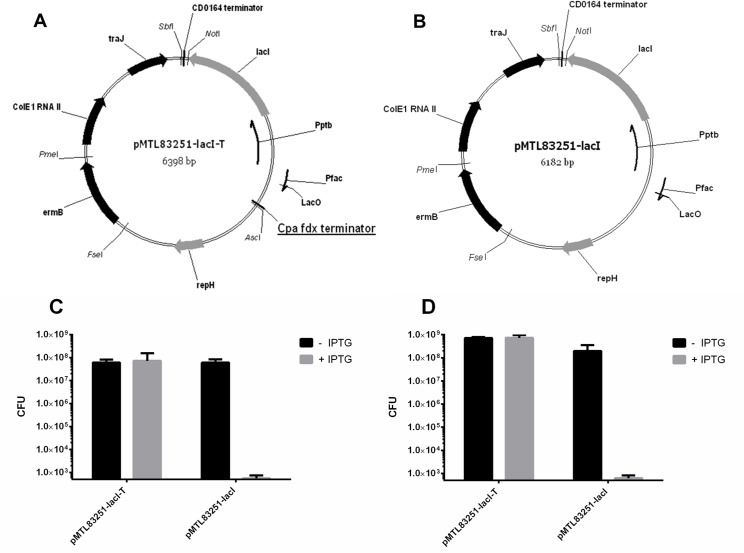
Plasmid maps and the conditionality analysis of the conditional replicon. Plasmid maps of the non-conditional control plasmid pMTL83251-lacI-T (**A**) and conditional plasmid pMTL83251-lacI (**B**). Key: CD0164 terminator, a transcriptional terminator isolated from downstream of the *Clostridium difficile* strain 630 CD0164 gene; *lacI*, the *E*. *coli* gene encoding LacI repressor; P**_ptb_**, the promoter of the *C*. *beijerinckii* gene encoding phosphotransbutyrylase; P**_fac_**, the promoter of the *C*. *pasteurianum* ferredoxin gene derivatised to include an *E*. *coli lac* operator; *repH*, replication region of the *Clostridium butyricum* plasmid pCB102; *ermB*, the macrolide-lincosamide-streptogramin B antibiotic resistance gene of plasmid pAMß1; ColE1, the replication origin of plasmid ColE1, and; *traJ*, transfer function of the RP4 *oriT* region. Cpa fdx terminator, transcriptional terminator of the ferredoxin gene of *C*. *pasteurianum* (this feature is underlined due to its existence only in **A**: pMTL83251-lacI-T, but not in **B**: pMTL83251-lacI). **C** and **D**: the ability to replicate of pMTL83251-lacI-T and pMTL83251-lacI in of *C*. *acetobutylicum* (**C**) and *C*. *sporogenes* (**D**) in the absence (CFU account in black) and presence (CFU account in grey) of IPTG.

Having established the functionality of the P_fac_ inducible promoter, the *cat* gene was deleted from plasmid pMTL83251-YZ2, in order to bring the pCB102 replicon [31] under the transcriptional control of P_fac_. Two plasmids were created. In the one (pMTL83251-lacI-T see Fig 2A), pMTL83251-YZ2 was cleaved with BamHI and HindIII, the sticky-ends created blunt-ended by treatment with T4 polymerase, and the resultant linear fragment subjected to self-ligation. In a second plasmid (pMTL83251-lacI, see Fig 2B), pMTL83251-YZ2 was digested with BamHI and AscI, the sticky-ends created blunt-ended by treatment with T4 polymerase, and the resultant linear fragment subjected to self-ligation.

To confine the conditional replicon to a cassette compatible with the pMTL80000 modular vectors series [32], the DNA fragment encompassing the P_fac_/P_ptb_::*lacI* cassette, plus the pCB102 replicon, was localised to a portable AscI-FseI fragment. This was accomplished by the following steps. In the first instance, the NotI site preceding the P_fac_/P_ptb_::*lacI* cassette of pMTL83251-lacI was changed to an AscI site using a QuikChange Site-Directed Mutagenesis Kit (Stratagene) and primers NotI/AscI and NotI/AscI-antisense, to yield the plasmid pMTL87250. This now allowed the excision of the P_fac_/P_ptb_::*lacI* cassette, together with the pCB102 replicon, as a 3537 bp AscI and FseI fragment. This was substituted in place of the equivalent restriction fragment encoding the pBP1 replicon in plasmid pMTL-SC1. The plasmid obtained was designated pMTL-YZ14. In parallel, pMTL-YZ13 was made replacing the pBP1 replicon in pMTL-SC1 with replicon pCB102.

**Fig 3:**
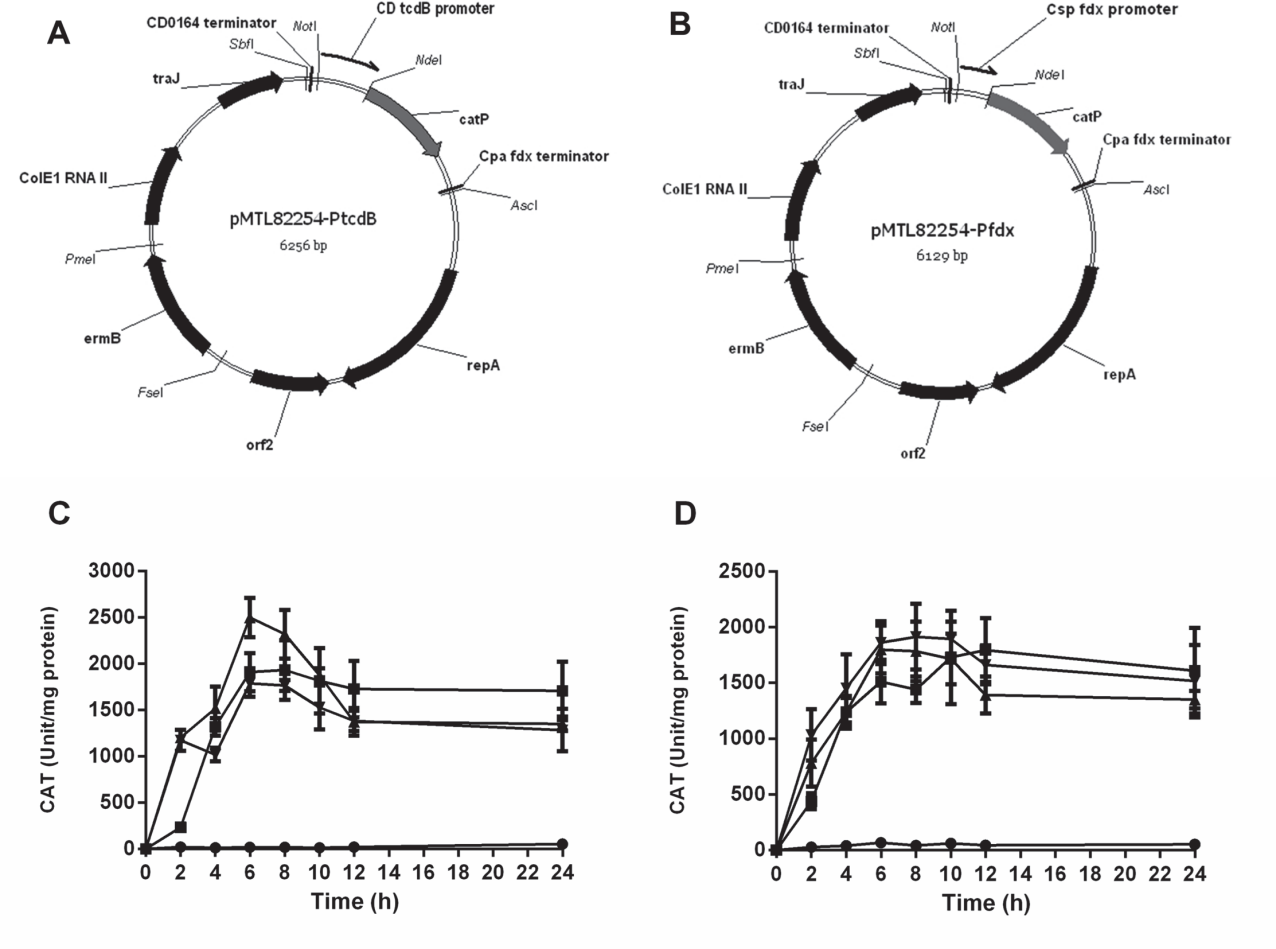
Analysis of the functionality of the exogenous TcdR in two clostridial strains (CRG3011 and CRG3817). Plasmid maps of the pMTL82254-P_**tcdB**_ (**A**) and pMTL82254-P_**fdx**_ (**B**). Key: CD0164 terminator, a transcriptional terminator isolated from downstream of the *C*. *difficile* strain 630 CD0164 gene; *catP*, a *C*. *perfringens*-derived gene encoding chloramphenicol acetyltransferase; Cpa fdx terminator, transcriptional terminator of the ferredoxin gene of *C*. *pasteurianum*; *repA* and *orf2*, replication region of the *C*. *botulinum* plasmid pBP1; *ermB*, the macrolide-lincosamide-streptogramin B antibiotic resistance gene of plasmid pAMß1; ColE1, the replication origin of plasmid ColE1, and; *traJ*, transfer function of the RP4 *oriT* region. CD *tcdB* promoter, the promoter region of the *C*. *difficile tcdB* gene; Csp fdx promoter: the promoter region of the *C*. *sporogenes fdx* gene. (**C)**: CAT activity of either *C*. *acetobutylicum* ATCC 824 wild type or CRG3011 (*tcdR* containing *C*. *acetobutylicum* ATCC 824) carrying plasmids pMTL82254-P_**tcdB**_ and pMTL82254-P_**fdx**_. (**D)**: CAT activity of either *C*. *sporogenes* NCIMB 10969 wild type or CRG3817 (*tcdR* containing *C*. *sporogenes* NCIMB 10969) carrying plasmids pMTL82254-P_**tcdB**_ and pMTL82254-P_**fdx**_. Black circles ●, wild type with pMTL82254-P_**tcdB**_; black squares ■, wild type with pMTL82254-P_**fdx**_; black triangles ▲, CRG3011/CRG3817 with pMTL82254-P_**tcdB**_; black triangles ▼, CRG3011/CRG3817 with pMTL82254-P_**fdx**_.

Plasmid pMTL82254-P_tcdB_ as shown in Fig 3A, was constructed by excising a ca. 334 bp NotI/NdeI fragment from the plasmid pMTL-SC1 [15] and inserting it between the NotI and NdeI sites of plasmid pMTL82254 [32]. For comparative purposes, a second plasmid was constructed identical to pMTL82254-P_tcdB_, but in which the fragment encompassing the P_tcdB_ promoter was replaced with an equivalent NotI/NdeI fragment encompassing the P_fdx_ promoter. This plasmid was designated pMTL82254-P_fdx_ as illustrated in Fig 3B.

### Integration of the *tcdR* gene into the clostridial host genome using ACE

A DNA fragment encompassing the *tcdR* structural gene encoding TcdR and its ribosome binding (RBS) site was PCR-amplified from the chromosome of the *C*. *difficile* strain 630 with primers tcdR-F and tcdR-R, and cloned into the vector pGEM-T. This localised the *tcdR* gene and RBS to a NotI-BamHI fragment which was excised and inserted between the equivalent sites of the plasmid pMTL-ME6C to yield the plasmid pMTL-ME6C-tcdR. Plasmid pMTL-ME6C is essentially plasmid pMTL-JH14, but which lacks the lacZ’- encoding region residing between the shorter Left Homology Arm (LHA, encoding a *pyrE* allele) and the longer Right Homology Arm (RHA, encoding CAC0028) and in which a region of DNA encompassing the transcriptional terminator of the *Clostridium pasteurianum* ferredoxin gene has been inserted 3’ to the *pyrE* allele, and preceding CAC0028.

**Fig 4:**
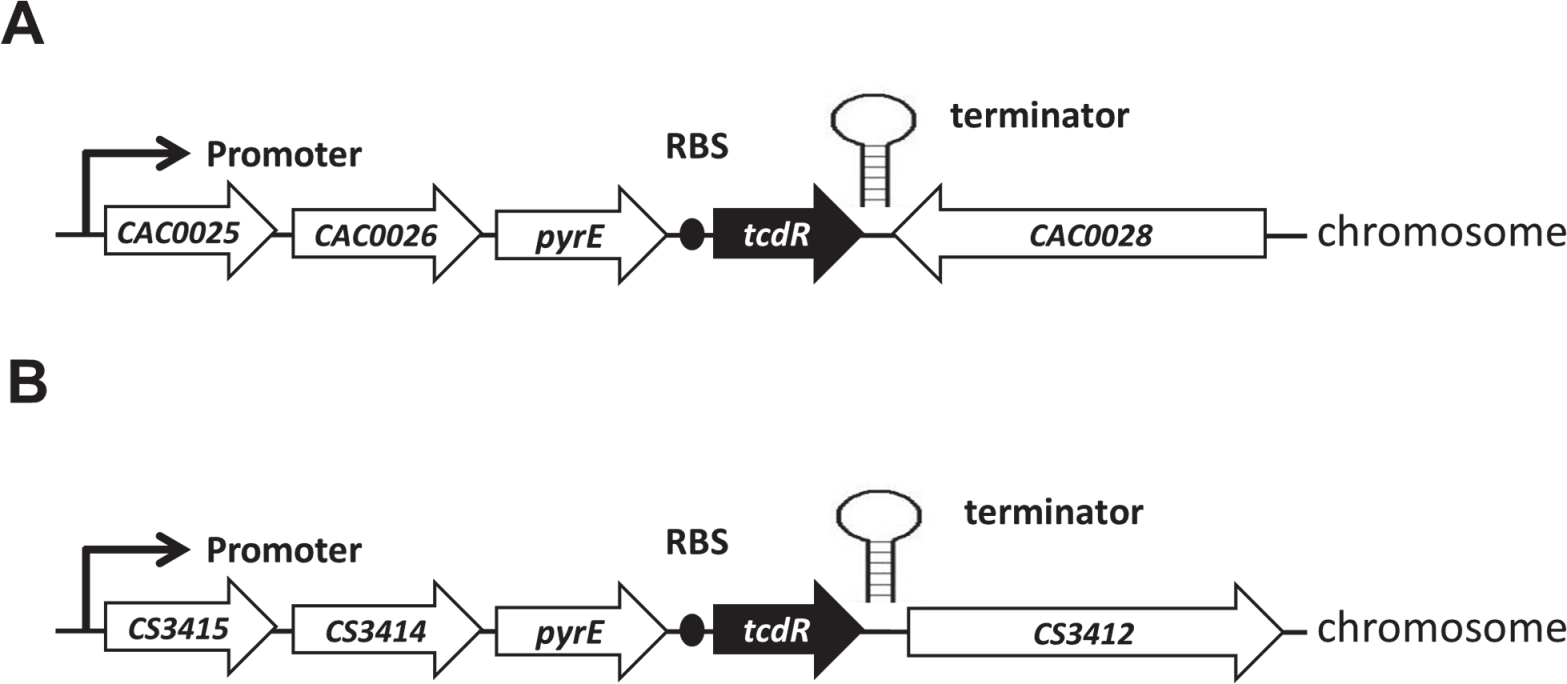
Schematic representations of the genome of *tcdR* strain of *C*. *acetobutylicum* (A) and *C*. ***sporogenes* (B).** A promoter-less copy of the *tcdR* gene (including its ribosome binding site, RBS) of *C*. *difficile* strain 630 has been inserted into the genome using ACE technology immediately downstream of the *pyrE* gene (**A**: CAC0027, **B**: CS3413). Illustrated are the surrounding genes, and the position of the promoter responsible for expression of t*cdR*, and the position of the *tcdR* RBS. The illustrated terminator is the T1 transcriptional terminator of the *C*. *pasteurianum* ferredoxin gene.

To introduce the *tcdR* gene into the chromosome of *C*. *acetobutylicum*, Allele-Coupled Exchange (ACE) was employed as described in Heap et al. [10]. *In vivo* methylated pMTL-ME6C-tcdR plasmid DNA was transformed into the ACE-generated *C*. *acetobutylicum pyrE* mutant essentially as described in Heap JT et al., 2012. Transformed cells were plated onto CGM agar supplemented with 15 μg/ml Tm and 20 μg/ml uracil. After 24 h, fast growing single colonies were picked and re-streaked twice onto CGM agar containing 15 μg/ml Tm and 20 μg/ml uracil. These cells represented those in which the plasmid had integrated into the genome by single cross-over recombination between the RHA. Thereafter, cells were streaked onto CBM agar to select for cells able to grow in the absence of exogenous uracil as a consequence of plasmid excision (through recombination between the duplicated LHA) and restoration of a functional *pyrE* allele. The final construct has the *tcdR* gene, together with its RBS, inserted immediately downstream of the *pyr*E gene, residing upstream of the *Clostridium pasteurianum* transcriptional terminator. The *tcdR* gene is transcribed from the promoter upstream of CAC0025 (as demonstrated in Fig 4A). The *C*. *acetobutylicum* strain generated was designated CRG3011.

To generate a strain producing functional TcdR protein in *C*. *sporogenes* NCIMB 10696, the following steps were undertaken. A DNA fragment encoding TcdR and its ribosome binding (RBS) site was excised as a NotI-BamHI fragment from plasmid pMTL-ME6C-tcdR, and inserted between the equivalent sites of the plasmid pMTL-YZ29 to yield the plasmid pMTL-YZ29-tcdR. ACE vector pMTL-YZ29 is as same as pMTL-JH29 with the Gram-positive replicon being changed to pCD6 instead of pIM13. Plasmid pMTL-YZ29-tcdR plasmid DNA was transformed into the ACE-generated *C*. *sporogenes pyrE* mutant described in Heap et al. [10]. Transformed cells were plated onto TYG agar supplemented with 15 μg/ml Tm. After 24 h, fast growing single colonies were picked and re-streaked twice onto TYG agar containing 15 μg/ml Tm. These cells represented those in which the plasmid had integrated into the genome by single cross-over recombination between the RHA. Thereafter, cells were streaked onto MACC minimal medium agar to select for cells able to grow in the absence of exogenous uracil as a consequence of plasmid excision (through recombination between the duplicated LHA) and restoration of a functional *pyrE* allele. The final construct has the *tcdR* gene, together with its RBS, inserted immediately downstream of the *pyrE* gene from where it is transcribed from the promoter upstream of CS3415 (Fig 4B). The strain generated was designated CRG3817.

### Chloramphenicol acetyltransferase (CAT) assay

CAT activity was determined according to the method of Shaw. [33]. A quartz cuvette was prepared containing 540 μl of 100 mM Tris buffer (pH 7.8), 200 μl of 2.5 mM DTNB (5,5′-dithiobis-2-nitrobenzoic acid) solution in 100 mM Tris buffer (pH 7.8), 200 μl of 5.0 mM freshly prepared acetyl Coenzyme A solution in deionized water and 10 μl of cell lysate. The cuvette was pre-warmed to 25°C, and the reaction initiated by adding 10 μl of 0.3% w/v Cm solution in deionized water. The initial rate of increase of absorption at 412 nm was measured using an Analytik Jena SPECORD 250 PLUS spectrophotometer.

### Fermentation

For *C*. *acetobutylicum*, batch fermentation experiments were carried out in 100 ml volumes of CBMS broth in 250 ml conical flasks. For *C*. *sporogenes*, batch fermentation experiments were carried out in 100 ml volumes of P2Y broth in 250 ml conical flasks. Cultures were inoculated to a starting OD_600_ of approximately 0.05 with an overnight starter culture, and incubated in an anaerobic cabinet at 37°C for 96 h. At each sample point, 1 ml samples were removed, placed on ice and centrifuged at 16,000 x *g* for 1.5 minutes. Supernatants were stored at -80°C before analysis by high performance liquid chromatography. OD_600_ was also measured at each time point. Samples subjected to heat shock (80°C, 10 min) were taken at time points 11, 24, 48, 72 and 96 h for sporulation assay.

### Analytical techniques

The supernatant samples described above were subjected to HPLC analysis using a Varian Prostar HPLC (model 240) equipped with an Aminex HPX-87H Ion Exclusion Column Column (300 x 7.8 mm Biorad), a refractive index detector (Varian 356LC) and a photo diode array (UV) detector (Varian). The mobile phase used was 0.005 M H_2_SO_4_ and the column was operated at 30°C with a flow rate of 0.6 ml/min. The injection volume was 20 μl and elution was isocratic. Supernatants were diluted appropriately in Milli-Q-water and filtered through a 0.2 μm pore size membrane filter prior to analysis.

### Isolation of transposon mutants

For *C*. *acetobutylicum*, plasmid pMTL-YZ14 was *in vivo* methylated and transformed into *C*. *acetobutylicum* CRG3011 as described previously [30]. The transformed cells were selected on CGM agar containing Em and were incubated for 48 to 72 h in order to expand the population of cells containing the transposon plasmid. All growth was harvested by flushing the whole plate with CGM broth with 10% Glycerol, and serial dilutions were made and plated onto CGM agars in duplicate: CGM agars without any antibiotics to calculate transposition frequency, CGM agars containing Tm (15 μg per ml) and IPTG (1 mM) to select for the transposon-based *catP* antibiotic marker and induce plasmid loss at the same time. Cell suspensions were stored at -80°C for future use. Individual colonies, visible after 12 to 16 h, were picked and patch plated onto CGM agars containing Tm only and CGM agars containing Em, respectively, to check for plasmid loss and for further analysis and/or phenotypic screening.

For *C*. *sporogenes*, plasmid pMTL-YZ14 was transformed into *C*. *sporogenes* CRG3817 strain as described previously without methylation. The transformed cells were selected on TYG agar containing Em and were incubated for 24 to 48 h. The plating and selections were performed essentially as described for *C*. *acetobutylicum* except for the growth medium employed.

## Results

### Transcription can affect maintenance of pCB102 replicon-based plasmids

During the construction of the ClosTron plasmid pMTL007 [30], it was noted that one of the derivative plasmids, pMTL540F, was unable to transform *C*. *acetobutylicum*. In contrast, a previously constructed plasmid pMTL540FT, transformed the same clostridial host at normal frequencies [34]. The only difference between the two plasmids is the presence of a transcriptional terminator, positioned between the strong P_fac_ promoter and the pCB102 plasmid replicon. We hypothesised that transcription into the replication region was interfering with the ability of the plasmid to stably establish itself in a transformed cell. It follows, that were the transcription from P_fac_ to be placed under regulatory control, and repressed, then the plasmid should be successfully installed in the transformed cell. Moreover, by adding the requisite inducer to a transformed cell, then the activation of transcription into the replicon region should subsequently lead to plasmid loss. To test this system, an inducible promoter system was required.

### Modularisation of an IPTG inducible promoter cassette

We have previously demonstrated that P_fac_ controlled expression of a *catP* gene in *C*. *acetobutylicum* and *C*. *sporogenes* could be repressed by the incorporation of an *E*.*coli lacI* gene onto a plasmid backbone in which expression of the repressor gene was placed under the transcriptional control of the P_ptb_ promoter of the *C*. *beijerinckii ptb* (phosphotransbutyrylase) gene. Moreover, repression could be relieved by the addition of exogenous IPTG, resulting in production of *cat-*encoded chloramphenicol acetyltransferase (CAT). For convenience, we elected to localise the P_ptb_::*lacI* element and P_fac_ promoter to a single portable restriction fragment. As the region encompassing these elements on the previously made plasmid, pMTL5401Fcat [30], were separated by a region of DNA encompassing *oriT*, we elected to delete this element during this process—*oriT* is superfluous as it is not needed for clostridia that can be transformed, and is in any case already part of the modular vector series (31) that we later planned to use. Accordingly, we used QuikChange to delete *oriT*, converted the P_ptb_::*lacI* + P_fac_ + *cat* region to a NotI/HindIII restriction fragment (Materials and Methods), and inserted it between the equivalent sites of plasmid pMTL83251 to yield plasmid pMTL-YZ2 (Fig 1A).

To confirm that expression of the *cat* gene remained under IPTG-inducible control, we transformed pMTL-YZ2 into *C*. *acetobutylicum* or *C*. *sporogenes*, and then grew the transformants in the presence or absence of added inducer. IPTG-mediated induction of CAT activity was observed in both organisms, but a significant basal level of expression was observed in *C*. *acetobutylicum* (Fig 1B) in the absence of IPTG, indicating the repression by LacI is less stringent than in *C*. *sporogenes*, where tight repression was evident (Fig 1C). This is consistent with a decrease in expression of *lacI* due to a reduced activity of the P_ptb_ promoter as cells entered the solventogenesis phase in *C*. *acetobutylicum* [35].

### Development of a conditional replicon

In order to test our hypothesis, that the establishment of a pCB102 replicon-based plasmid could be controlled by the IPTG inducible promoters system, we constructed two new plasmids, pMTL83251-lacI-T and pMTL83251-lacI (Fig 2A and 2B). The essential difference between the two plasmids is that pMTL83251-lacI-T carries a transcriptional terminator (that of the ferredoxin gene of *Clostridium pasteurianum*) between the P_fac_ promoter and the *repH* replication gene of the pCB102 replicon. Plasmid pMTL83251-lacI is broadly equivalent to pMTL-YZ2, except it lacks the *cat* gene.

Transformed cells of *C*. *acetobutylicum* ATCC 824 carrying either plasmid were cultivated and the effect of adding IPTG on plasmid retention estimated. Specifically, *C*. *acetobutylicum* transformants were cultured for 12 h in CGM broth supplemented with Em to select for the plasmid. The culture was then washed twice in PBS to remove the antibiotic and then used to inoculate fresh, CGM broth with and without IPTG (1mM) at 1% (vol/vol). After 12 h of growth, the culture was plated to enumerate Em^R^ Colony Forming Units (CFU). As predicted, plasmid pMTL83251-lacI was found to only be stably maintained in the absence of IPTG (Fig 2C). In the presence of IPTG, the plasmid was rapidly lost from the cell population (Fig 2C), as evidenced by an almost complete loss of CFU on plates supplemented with Em. A similar loss was not evident in cells harbouring pMTL83251-lacI-T (Fig 2C). It was concluded that transcriptional read-through into the pCB102 replication region was interfering with plasmid replication/ maintenance. One explanation for this phenomenon might have been that the RepH protein was being overexpressed, and that over production of RepH was detrimental to the cell. However, no difference in growth rate was observed between cells carrying pMTL83251-lacI cultivated in medium in the presence or absence of IPTG (data not shown). To test whether a similar phenomenon was observed in a different clostridial species, the equivalent experiment was replicated in *C*. *sporogenes* NCIMB 10696, in this case using TYG broth. A similar result to that seen in *C*. *acetobutylicum* was obtained. Thus, in the presence of IPTG, plasmid pMTL83251-lacI was rapidly lost from the population (Fig 2D), whereas retention of plasmid pMTL83251-lacI-T was unaffected by addition of the inducer (Fig 2D). These data indicate that the system has broader application than just a single *Clostridium* species.

### Endowment of the genome with *tcdR*

Having derived a prototype conditional delivery vehicle for use in *C*. *acetobutylicum* and *C*. *sporogenes* we sought to implement our *mariner* transposon system in these clostridia by endowing them with a genomic copy of the *C*. *difficile tcdR* gene. The presence of TcdR in either clostridia would direct the expression of the transposase gene under the control of the P_tcdB_ promoter when introduced on a plasmid. Accordingly, a promoter-less copy of the *tcdR* gene was cloned into the requisite ACE vectors needed to both convert the *pyrE* allele of the clostridial *pyrE* mutant hosts used back to wild type using the ACE protocol (Materials and Methods), while simultaneously inserting the *tcdR* gene into the genome immediately downstream of the restored *pyrE* gene (Fig 4A). This placed the *tcdR* gene under the control of the promoter responsible for *pyrE* transcription. The strain of *C*. *acetobutylicum* carrying *tcdR* in its genome was designed CRG3011, whereas the equivalent strain of *C*. *sporogenes* was designated CRG3817 (Fig 4B).

To test the functionality of TcdR in the two clostridial strains (CRG3011 and CRG3817), the P_tcdB_ or the P_fdx_ promoter were inserted upstream of the promoter-less *catP* gene of the reporter plasmid pMTL82254, to yield the respective plasmids pMTL82254-P_tcdB_ and pMTL82254-P_fdx_ (Fig 3A and 3B). Each plasmid was introduced into both the wild type strains of *C*. *acetobutylicum* and *C*. *sporogenes*, and their respective derivatives (CRG3011 and CRG3817) carrying *tcdR*. Subsequently, the four cell lines were cultivated in CGM or TYG broth, cells harvested at designated time points and lysates prepared for assays of CAT activity (Fig 3C and 3D). In both organisms it was evident from the low CAT activity observed in the wild type cell lines that the P_tcdB_ promoter was essentially inactive in the absence of TcdR. In contrast the level of CAT expression seen in CRG3011 and CRG3817 cells carrying pMTL82254-P_tcdB_ was broadly equivalent to that seen in cells carrying plasmid pMTL82254-P_fdx_, where expression was due to the strong P_fdx_ promoter.

It was of interest to determine whether the presence of *tcdR* in the genome of either organism had any discernible effect. It was evident that in either case, there were no detectable alterations to either clostridial species in terms of appearance under the microscope, colony morphology, sensitivity to heat shock, growth rate in liquid media or the solvent/acid supernatant profiles observed after four days growth of the cells (S1, S2 and S3 Figs).

### Use of the conditional vector for transposon delivery

**Fig 5:**
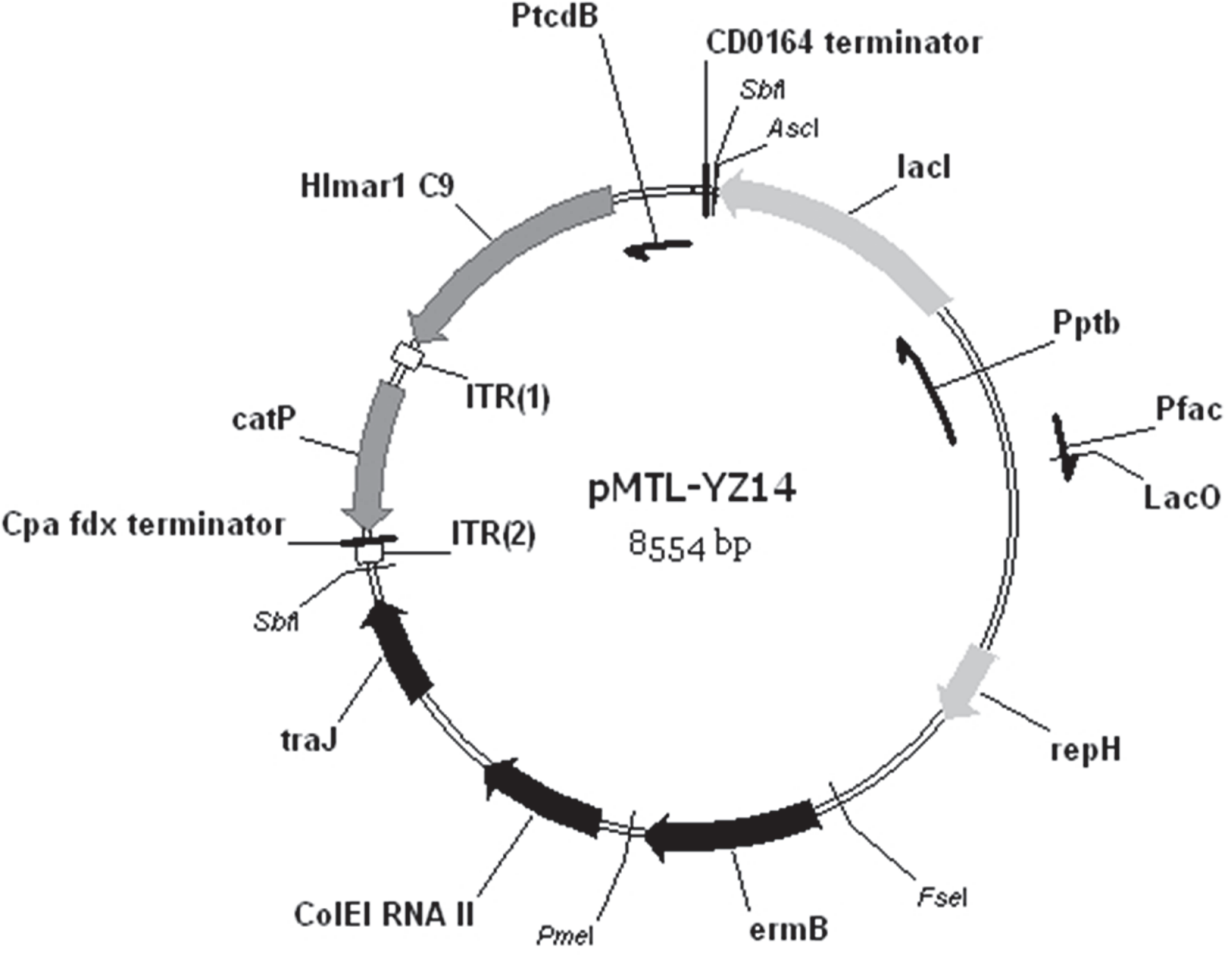
Vector map of plasmid pMTL-YZ14. Expression of the hyperactive *mariner* transposase gene *Himar1 C9* was driven by the *C*. *difficile* toxin B promoter, P_tcdB_. The plasmid backbone consisted of the conditional replicon between restriction sites AscI and FseI, the macrolide-lincosamide-streptogramin B antibiotic resistance gene *ermB*, and the Gram-negative replicon, ColE1. The whole mariner element (i.e., transposase gene and *catP* mini-transposon) can be excised as a SbfI fragment. The control plasmid pMTL-YZ13 was identical, except that the Gram-positive replicon is the pCB102 replicon from *C*. *butyricum*. This plasmid conforms to the pMTL80000 modular system for *Clostridium* shuttle plasmids [32].

By endowing the two clostridial hosts with *tcdR*, effective transposition of the mini-transposon from plasmid pMTL-SC1 should now occur. Before testing this assumption, the plasmid was first modified to incorporate the pCB102 conditional replicon in place of the pBP1 replication region. This was accomplished by substituting the modular pBP1 replicon, excised as an AscI and FseI restriction fragment, with an equivalent fragment encompassing the modular, conditional pCB102 replicon of pMTL87250. The plasmid generated was designated pMTL-YZ14 (Fig 5). To test its effectiveness as a transposon delivery system, the plasmid was transformed into the *tcdR*-containing strains of *C*. *acetobutylicum* and *C*. *sporogenes*, CRG3011 and CRG3817. Transfer of the plasmid was initially selected on solidified media supplemented with Em and left on plates for 24 (CRG3817) to 72 (CRG3011) hours, before being plated on media containing Tm and IPTG to select for transposon events and promote plasmid loss, respectively. After 12 to 16 h of incubation, Tm ^R^ colonies were visible at a frequency of 2.6 (±0.6) ×10^–4^ and 3.2 (±0.5) ×10^–4^ (calculated as the ratio of Tm ^R^ CFU to total CFU) in *C*. *acetobutylicum* and *C*. *sporogenes*, respectively. A total of 100 Tm ^R^ colonies of each *Clostridium* species were picked and patch plated onto appropriate solidified rich media containing either Tm or Em to estimate the percentage of cells that had lost the plasmid. In total, 80% of *C*. *acetobutylicum* and 100% of *C*. *sporogenes* colonies were Tm resistant (^R^) and Em sensitive (^S^), indicative of successful insertion of the *catP* mini-transposon into chromosome and substantive plasmid loss.

**Fig 6:**
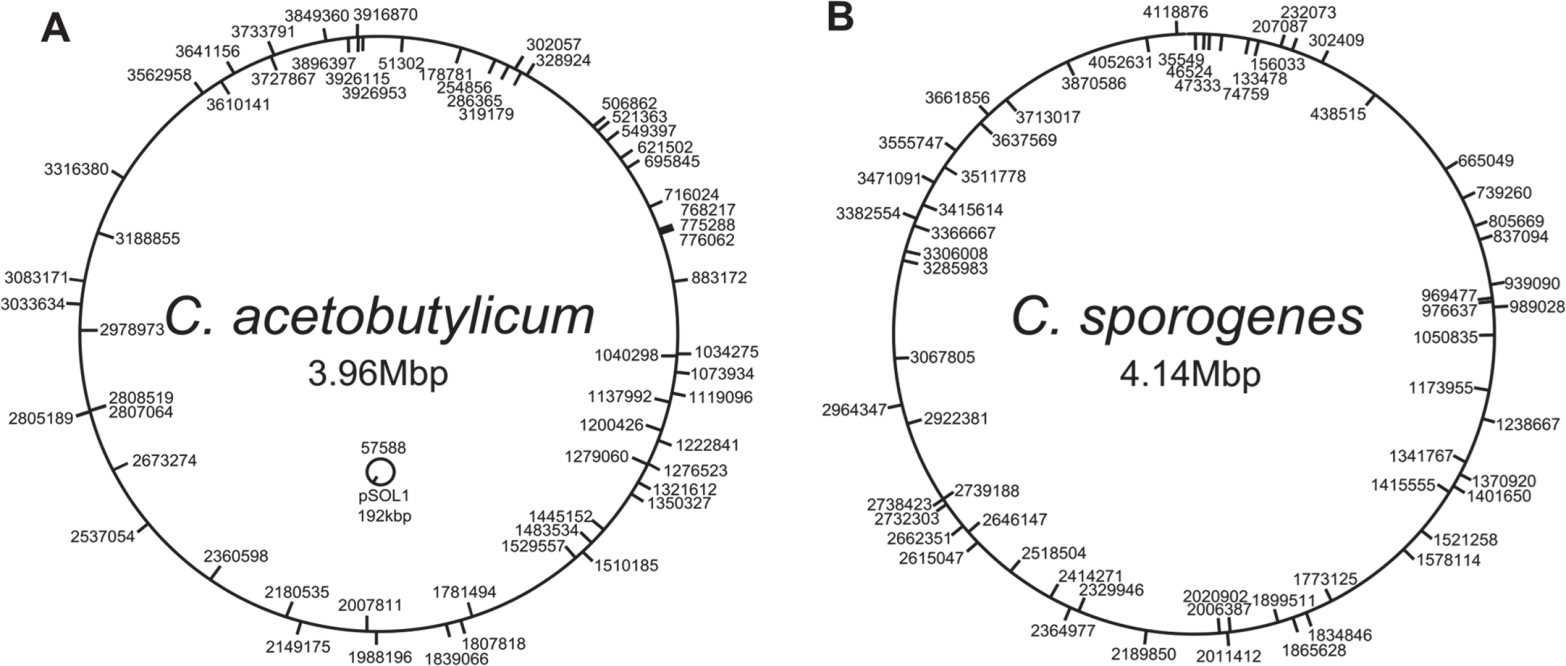
Genetic map of *mariner* transposon insertions in *C*. ***acetobutylicum* (A) and in *C*. *sporogenes* (B).** Sixty independent transposon mutants were identified and the insertions were sequenced. Insertions in the plus orientation are marked on the circle exterior. Insertions in the minus orientation are marked on the circle interior. Numbers indicate the precise point of insertion according to genome sequence data for (**A**) *C*. *acetobutylicum* ATCC 824 genome and megaplasmid pSOL1 (Refseq number NC_003030.1 and NC_001988.2; GenBank accession number AE001437 and AE001438) [28] and (**B**) *C*. *sporogenes* NCIMB 10956 (GenBank accession number CP009225).

To establish whether transposition had indeed occurred, a more thorough molecular characterisation was undertaken. Genomic DNA of 60 randomly selected Tm ^R^ and Em ^S^ clones was prepared, digested with HindIII and subjected to Inverse PCR after self-ligation. The gel-purified inverse PCR products were subjected to nucleotide sequence analysis using the primer catP-INV-R2 to determine the sites of insertion. The data demonstrated that each transposon insertion had taken place at a different position around the genome as illustrated in Fig 6.

For *C*. *acetobutylicum* (Fig 6A), all of the Tm ^R^ clones sequenced had a single transposon insertion, with the exception of one which had a double insertion revealed by Inverse PCR (S4 Fig). In one clone the mini-transposon had an insertion into the megaplasmid pSOL1. The remainder had all inserted at random around the chromosome. In total, there were 34 insertions in the plus strand and 27 in the minus strand, with 51 of the insertions located within open reading frames. In the case of *C*. *sporogenes* (Fig 6B), two out of the sixty clones contained double insertions of the mini-transposon. The remaining 58 clones all contained single insertions scattered at random around the genome. In total, there were 30 insertions in the plus strand and 32 in the minus strand. The number of insertions within open reading frames was 49, representing a slightly higher ratio of insertions into intergenic regions compared to *C*. *acetobutylicum*.

As a control, both clostridial strains were also transformed with pMTL-YZ13, which is identical to pMTL-YZ14 except that the pCB102 replicon is not preceded by the IPTG inducible cassette (P_ptb_::*lacI*::P_fac_). In this case, following growth on plates supplemented with Em, cells were plated on CGM agar containing Tm, but no IPTG. A total of 24 Tm ^R^ colonies of each strain were picked and re-streaked 3 times, one more passage than described in Cartman and Minton. [15] Colonies were then patch plated onto solidified media with and without Em to estimate plasmid loss. Only 4 of clones had become Em ^S^ in the case of *C*. *acetobutylicum* and just 3 out of 24 in the case of *C*. *sporogenes*. These data underlined the advantages of using the conditional pCB102 replicon.

### Isolation of phenotypic mutants affected in sporulation/ spore germination

To demonstrate the utility of the developed system for forward genetic studies, we screened representative transposon libraries of the two clostridial species for mutants with defects in the ability to form colony forming units (CFUs) after heat shock. These were presumed to be defective in either the process of sporulation or spore germination, ie., spo/ger¯ mutants. The number of Tm ^R^ clones screened for *C*. *acetobutylicum* and *C*. *sporogenes* were approximately 3000 and 6000, respectively. Clones were inoculated into fresh CBM or YTG liquid broth (*C*. *acetobutylicum* and *C*. *sporogenes*, respectively) in replica 96-well microtitre plates and anaerobically incubated for one week to allow sporulation to take place. The microtitre plates were subjected to heat treatment (80°C for 10 min), while the other was held at room temperature. The contents of each microtitre well were individually plated onto either CGM (*C*. *acetobutylicum*) or YTG (*C*. *sporogenes*) agar media supplemented with Tm. Those cells unable to form colonies after heating 80°C for 10 min, but still able to produce colonies when not subjected to heat shock, were deemed possible sporulation or germination mutants.

In *C*. *acetobutylicum* a single transposon mutant was isolated with the desired spo/ger¯ phenotype, whereas in *C*. *sporogenes*, two such mutants were obtained. Inverse PCR was carried out on the isolated genomic DNA of the three mutants to identify the genes which had been inactivated (Table 3). In the case of *C*. *acetobutylicum*, the mini-transposon was found to have inserted in a gene (CAC2631) encoding a hypothetical protein that exhibited homology (70% similarity in DNA sequence) to the *Bacillus subtilis* spore protein YkuD reported to be involved in assembly of proteins on the forespore [36]. The two genes affecting the ability of *C*. *sporogenes* to form CFUs after heat shock were CLSPO_c23320, a flavodoxin oxidoreductase, and CLSPO_c31790, annotated as encoding the stage V sporulation protein SpoVAD. The *spoVA* operon in *B*. *subtilis* encodes six proteins, each of which has several putative membrane-spanning domains, and together they have been suggested to be involved in dipicolinic acid DPA transport into the developing forespore and the release of Ca^2+^-DPA and other small molecules during spore germination [37,38]. The defect in this *C*. *sporogenes* gene is therefore consistent with the observed phenotype.

**Table 3:** Transposon insertion sites.

Strain	Phenotype	Transposon insertion site^a^	Location in genome (nt)^b^	ORF interrupted^c^	Description
*C*.*acetobutylicum* ATCC 824	spo/ger¯	ATACTAAACTTGATATTA**TA**—Tn—**TA**AATATAACTTTCTTCTTT	2740454	CAC2631	hypothetical protein(L,D-transpeptidase catalytic domain protein; YkuD (spore protein of Bacillus subtilis))
*C*.*acetobutylicum* ATCC 824	Auxotroph	CTAAATCATTTGCAAGAA**TA**—Tn—**TA**CACAAGGCTAATCTAATC	1119096	citB (CAC0971)	aconitate hydratase / dehydrogenase Catalyzes the conversion of citrate to isocitrate
*C*. *sporogenes* NCIMB 10696	spo/ger¯	ATTGCACTCTAATGGAAA**TA**—Tn—**TA**GAATAATATCTATAATAG	2615047	CLSPO_c23320	flavodoxin oxidoreductase
*C*. *sporogenes* NCIMB 10696	spo/ger¯	CAGCGCATAGCATATCTA**TA**—Tn—**TA**TCTGTATCCTTTAGATTA	3471091	spoVAD(CLSPO_c31790)	stage V sporulation protein AD
*C*. *sporogenes* NCIMB 10696	Auxotroph	ACTGTAAGTTTTACTATG**TA**—Tn—**TA**AGTACCAGGATCTTTATA	1834846	colA(CLSPO_c16750)	microbial collagenase
*C*. *sporogenes* NCIMB 10696	Auxotroph	CTACTACTAAATTATTTC**TA**—Tn—**TA**TATCTACTAGTAACTGGA	2364977	CLSPO_c21170	hypothetical protein
*C*. *sporogenes* NCIMB 10696	Auxotroph	ATTGTACTGCCATAGAAA**TA**—Tn—**TA**AATATAATACTCAAACTA	3285983	CLSPO_c29860	ABC-2 family transporter protein

^*a*^ The Tn insertion is indicated by dashes on either side, and the target site duplication is shown in boldface.

^*b*^ nt, nucleotide.

^*c*^ ORF, open reading frame.

The phenotype associated with the mutation in CLSPO_c23320 is less clear. Oxidoreductase is commonly related to aerotolerance in anaerobic organisms, providing protection against oxidative stress [39]. Since spores are important in the dissemination of the clostridial species it is likely that proteins with ability to scavenge the oxidative radicals are present in the outermost layers of the spores, and play a dual role—one in structural built-up of spores coat layers by mediating dityrosine cross-linking among proteins and/or second in resistance against oxidative stress [40,41] or they may have some other hitherto unknown function.

### Isolation of auxotrophic phenotypic mutants

To screen for auxotrophic mutants, the two libraries of Tm ^R^ colonies were simply replica plated onto either solidified rich media (CGM for *C*. *acetobutylicum* and YTG for *C*. *sporogenes*) or an appropriate minimal medium agar medium (P2 for *C*. *acetobutylicum* and MACC for *C*. *sporogenes*), in all cases supplemented with Tm.

A single auxotrophic mutant of *C*. *acetobutylicum* was isolated that failed to grow on P2 agar medium after 7 days of incubation at 37°C. Nucleotide sequencing of isolated genomic DNA demonstrated that the min-transposon insertion had inserted into CAC0971 (CitB), which is annotated as encoding aconitase (aconitate hydratase). In aerobic bacteria this enzyme is responsible for the conversion of citrate to iso-citrate as part of the tricarboxylic acid (TCA) cycle. Iso-citrate is further converted into the TCA cycle intermediates oxalosuccinate and α-ketoglutarate, and the later into the non-TCA cycle metabolite glutamate, which can subsequently be converted into arginine and glutamine. Whilst strict anaerobes are not generally regarded as being in the possession of a TCA cycle, two independent research groups have recently suggested that *C*. *acetobutylicum* possesses a complete, albeit bifurcated TCA cycle in which oxaloacetate flows to succinate both through citrate/α-ketoglutarate and via malate/ fumarate [42,43].

The gene coding for citrate synthase has been tentatively identified as CAC0970 and is the first gene of a putative tricistronic operon, comprising citrate synthase (CAC0970), aconitase (CAC0971) and isocitrate dehydrogenase (CAC0972), which is expressed at higher levels at stationary phase [43]. Accordingly, the mutant, together with *C*. *acetobutylicum* CRG3011, was plated onto P2 minimal agar medium, individually supplemented (1 mM) with the tricarboxylic and amino acids, citrate, iso-citrate, α-ketoglutarate, glutamate, glutamine, proline and arginine (Table 4). Qualitative growth of the mutant was only fully restored to wild type by exogenous glutamate, whereas growth was partially restored by glutamine. Intriguingly, the mutant remained unable to grow on the minimal media when supplemented with α-ketoglutarate, which is suggested to be the product of the conversion of iso-citrate by isocitrate dehydrogenase (CAC0971) and the substrate for the synthesis of glutamate [42]. Our results clearly implicate CAC0971 in glutamate biosynthesis, but may suggest that glutamate is derived from a source other than α-ketoglutarate, assuming that this ketone can be taken up by *C*. *acetobutylicum*. Whilst glutamine is most likely derived from glutamate, the fact that it could not entirely restore the growth of the *citB* mutant to wild type levels suggest that the reversal of the glutamate to glutamine reaction is not thermodynamically favourable under normal laboratory conditions.

**Table 4:** Characterization of the found auxotroph mutant with different additives in P2 minimal medium.

Supplementation	citB (CAC0972) mutant	*C acetobutylicum* CRG3011
Citrate	**˗˗**	++
Iso-citrate	**˗˗**	++
α-Ketoglutarate	**˗˗**	++
Glutamate	++	++
Glutamine	+	++
Proline	**˗˗**	++
Arginine	**˗˗**	++
Without supplementation	**˗˗**	++

No growth(˗˗), slight growth(+) and normal growth(++).

As a proteolytic clostridia, *C*. *sporogenes* is not able to undertake the biosynthesis of as many amino acids as the saccharolytic *C*. *acetobutylicum*. It was for this reason that a larger library was screened. In total, three mutants were isolate which were compromised in their ability to grow on minimal media compared to the wild type control. The inactivated genes were CLSPO_c16750, encoding a collagenase (annotated as ColA), CLSPO_c21170, coding for a hypothetical protein and CLSPO_c29860, annotated as an ABC-2 family transporter protein. A BLAST search of the protein sequence of CLSPO_c21170 revealed no putative conserved domains. The role of this protein in supporting growth on the minimal media employed, therefore, remains unknown. Similarly, the function of the protein encoded by CLSPO_c29860 is unclear, although the annotated name of the ABC-2 family of transporter proteins indicates it is likely involved in the translocation of an as yet unknown substrate(s)/ nutrient(s) into the cell. In the case of ColA, whilst there is no direct evidence to suggest bacterial *colA* genes are essential for survival and growth in defined medium, it is interesting to note that some of the organisms shown to elaborate collagenolytic enzymes are asaccharolytic [44]. Since their metabolism is dependent on the uptake of small peptides and amino acids, the production of collagenolytic enzymes by these organisms may be essential for survival and growth. Closely related with *C*. *botulinum*, *C*. *sporogenes* is proteolytic and asaccharolytic, and extra yeast extract is known to be essential for growth in fermentation compared to other saccharolytic clostridia. Hence, ColA could play an important role for the uptake of small peptides and amino acids in *C*. *sporogenes*.

## Discussion

In the current study, we have implemented two fundamental refinements to an existing *mariner* transposon system that greatly increase its utility for use in *Clostridium* spp. On the one hand we have devised an improved transposon delivery vehicle that is conditional for plasmid maintenance. This significantly reduces the background of antibiotic resistant transposon colonies that still retain the plasmid after plating under the non-permissive condition. On the other hand, we have devised a strategy whereby the benefits of using the P_tcdB_ promoter, which does not function in the *E*.*coli* donor but is confined to the target *Clostridium* cell, can be implemented in potentially any *Clostridium* species. This is simply achieved through the introduction of the cognate sigma factor gene, *tcdR*, into the target host genome using the well established gene integration technology, ACE.

The non-permissive condition for the maintenance of the plasmid carrying the conditional pCB102-based replicon in the clostridial cell is the presence of IPTG. The absence of IPTG represents the permissive condition. The molecular basis of plasmid loss in the presence of IPTG is currently unknown. The fact that transcriptional terminators positioned between the strong P_fac_ promoter and the pCB102 replicon prevents plasmid loss clearly indicated that the cause of this loss is increased transcription of the pCB102 replicon region. The replicative mechanisms used by the pCB102 replicon is currently unknown, save that the minimal replication region carries a single putative ORF designed RepH. It seems unlikely, however, that this is a consequence of overproduced RepH protein causing a metabolic burden on cells carrying the plasmid, as no growth rate difference is evident in cells carrying the plasmid in the presence or absence of IPTG. Moreover, it is still not certain that RepH actually encodes a protein. The predicted amino acid sequence has no functional homologue in current databases, and its primary sequence is highly unusual in being composed of 7 Arg and 22 Lys residues as well as 10 Cys residues from a total of only 104 amino acids. It may be that the replicative mechanism is RNA based, and that overproduction of RNA components leads to a breakdown in the ability of the plasmid to maintain itself in the cell, either by impeding replication or causing some form of catastrophic instability. Further work would be required to clarify the situation. In the meantime, in the absence of cause, the effect can be used to our advantage.

It should be noted that this conditional system as it stands cannot be used in an organism in which the IPTG inducible systems does not function. Thus, while we have shown that the conditional replicon functions effectively in *C*. *beijerinckii* (G. Little, unpublished), *C*. *botulinum* (C. Humphreys, unpublished) and *C*. *autoethanogenum* (C. woods, unpublished), it cannot be used in *C*. *difficile* (M. Fit, unpublished). The latter observation is because *C*. *difficile* apparently does not take up IPTG. However, it is clear that the IPTG inducible promoter system developed here can be substituted for with analogous inducible system shown to work in the target organism. Examples include the anhydrotetracycline inducible system exemplified in *C*. *difficile* [45], as well as the lactose inducible system of *C*. *perfringens* [46].

The second technical innovation described here is to capitalise on the conditional nature of the *C*. *difficile* P_tcdB_, in respective of being active only in the target host, and not the *E*. *coli* donor. This desirable feature prevents transposon activity in *E*. *coli* prior to transfer of the vector. To researchers unfamiliar with ACE technology, transferring *tcdR* into the clostridial host may seem not seem at first glance a trivial undertaking. However, the ACE method is extremely efficient, requiring the initial selection of the required *pyrE* uracil auxotrophic mutant on the basis of resistance to fluoroorotic acid (FOA), and thereafter, the integration of the *tcdR* gene concomitant with restoration of the *pyrE* allele to wild type, selecting for growth on minimal media lacking uracil. The interconversion of wild type and mutant *pyrE* alleles has now been accomplished in our laboratory using ACE in eight different clostridial species to date (unpublished data). Our data also shows, that at least in *C*. *acetobutylicum* and *C*. *sporogenes*, the presence of the *tcdR* gene has no discernible effect on the organisms’ phenotype. In any case, transposon mutagenesis should be very much viewed as a screening tool. Thus, it would expected that once the preliminary phenotype of a specific mutant has been determined, that a definitive mutation would be made in a clean background lacking *tcdR* using a directed method, such as the ClosTron or alternative allelic exchange procedures.

In the current study, the system was exemplified in *C*. *acetobutylicum* and *C*. *sporogenes*. Transposition into clostridial genome occurred at a frequency of 2.6 (±0.6) ×10^–4^ and 3.2 (±0.5) ×10^–4^ in C. *acetobutylicum* and *C*. *sporogenes* respectively, in a random fashion, with simultaneously plasmid loss at 80% and 100%, generating transposon mutants with just single insertion in an overwhelming majority of cases (98.3% and 96.7% in this study). Moreover, 51 of the 61 insertions sequenced (83.6%) in C. *acetobutylicum* and 49 of the 62 (79%) in *C*. *sporogenes* were located within protein coding sequences. This is within the range that would be expected for a random mutagen in *Clostridium*, considering that around 80% of the clostridial genome is protein coding.

We further demonstrated that the tool could be used to isolate specific mutants with a particular phenotype, by screening for clones affected in sporulation/germination as well as autotrophic strains which could no longer grow on minimal media. Our intention was merely to exemplify that the method could be used for such a purpose, rather than gather fundamental information on the nature of the cellular components identified. Nevertheless, some preliminary conclusions can be made on the nature of the mutants identified. Thus, in the main, the sporulation/germination mutants obtained were in genes that encoded proteins associated with *B*. *subtilis* spores (spore protein YkuD and SpoVA). The reason for the absence of CFU after heat shock when the *C*. *sporogenes* CLSPO_c23320 gene was inactivated (encoding a putative flavodoxin oxidoreductase) was less clear. Auxotrophic mutant screening has clearly implicated the *C*. *acetobutylicum* gene CAC0971 (encoding CitB, aconitase) in glutamate biosynthesis, and most likely glutamine, but has called in to question whether glutamate is formed from α-ketoglutarate. In the case of *C*. *sporogenes*, the reason why inactivation of the three genes (CLSPO_c16750, a collagenase; CLSPO_c21170, a hypothetical protein, and; CLSPO_c29860, a protein annotated as an ABC-2 family transporter protein) prevented growth on a minimal media were largely unclear, with the possible exception of the collagenase where a possible role in the uptake of small peptides and amino acids may exist.

## Conclusions

We have developed a novel *mariner* transposon system and exemplified it in *C*. *acetobutylicum* and *C*. *sporogenes*, where a number of auxotrophic and spore-related mutants were isolated. The system makes use of a conditional plasmid delivery vehicle, which may be deployed to significantly reduce the background of cells that still retain autonomous plasmids, as well as a conditional expression system that confines production of the *mariner* transposase to the target clostridial species, and not the *E*. *coli* donor. The system is able to generate completely random mutant libraries at high frequencies and should prove widely applicable to members of the genus *Clostridium*.

## Supporting Information

S1 Fig Sporulation profiles of (A) *C*. ***acetobutylicum* wild type and CRG3011; (B) *C*. *sporogenes* wild type and CRG3817 over 96 hours.** ClosTron mutants Cac-spoOA::CTermB and Cspo-spoOA::CTermB were included as a negative control.
(TIF)

S2 Fig Growth curves and fermentation profiles of *C*. *acetobutylicum* wild type (black circles) and CRG3011 (black squares).
(TIF)

S3 Fig Growth curves and fermentation profiles of *C*. *sporogenes* wild type (black circles) and CRG3817 (black squares).
(TIF)

S4 Fig Inverse PCR screens of nine random selected pMTL-YZ14 derived Tm ^R^ and Em ^S^ clones of CRG3011. Genomic DNA prepared from each clone was screened for the transposon based insertion. Lane M, 1kb ladder (NEB); lane-, negative control (genomic DNA of CRG3011); lane 1–9, pMTL-YZ14 derived Tm ^R^ and Em ^S^ clones 1 to 9, clone 3 shows double transposon insertion while the other clones have single insertion.
(TIF)
