# Supplementary figures and images for: A Universal Mariner Transposon System for Forward Genetic Studies in the Genus Clostridium
Source: PLoS One. 2015 Apr 2;10(4):e0122411. doi: 10.1371/journal.pone.0122411 (PMC4383383; doi:10.1371/journal.pone.0122411)

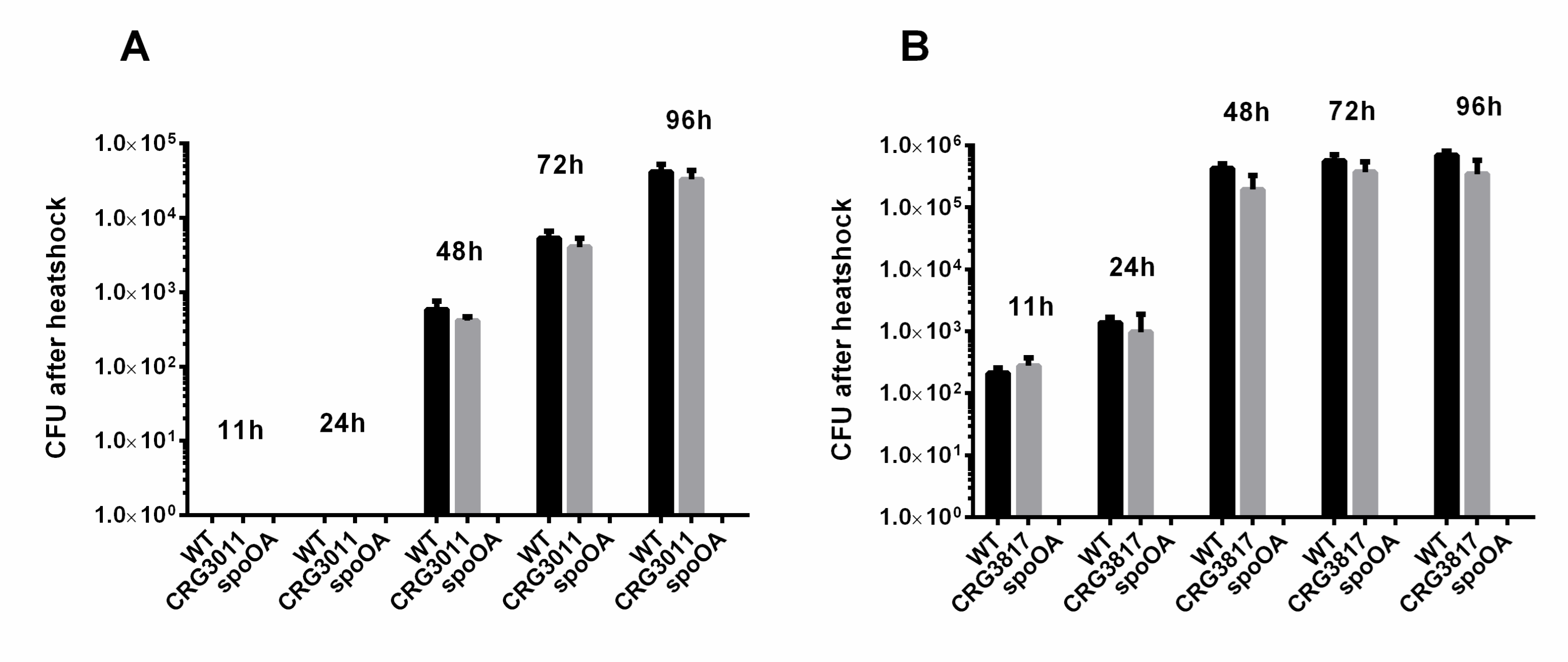

Supplement: S1 Fig — acetobutylicum wild type and CRG3011; (B) C. sporogenes wild type and CRG3817 over 96 hours. ClosTron mutants Cac-spoOA::CTermB and Cspo-spoOA::CTermB were included as a negative control. (TIF) [file pone.0122411.s001.tif]

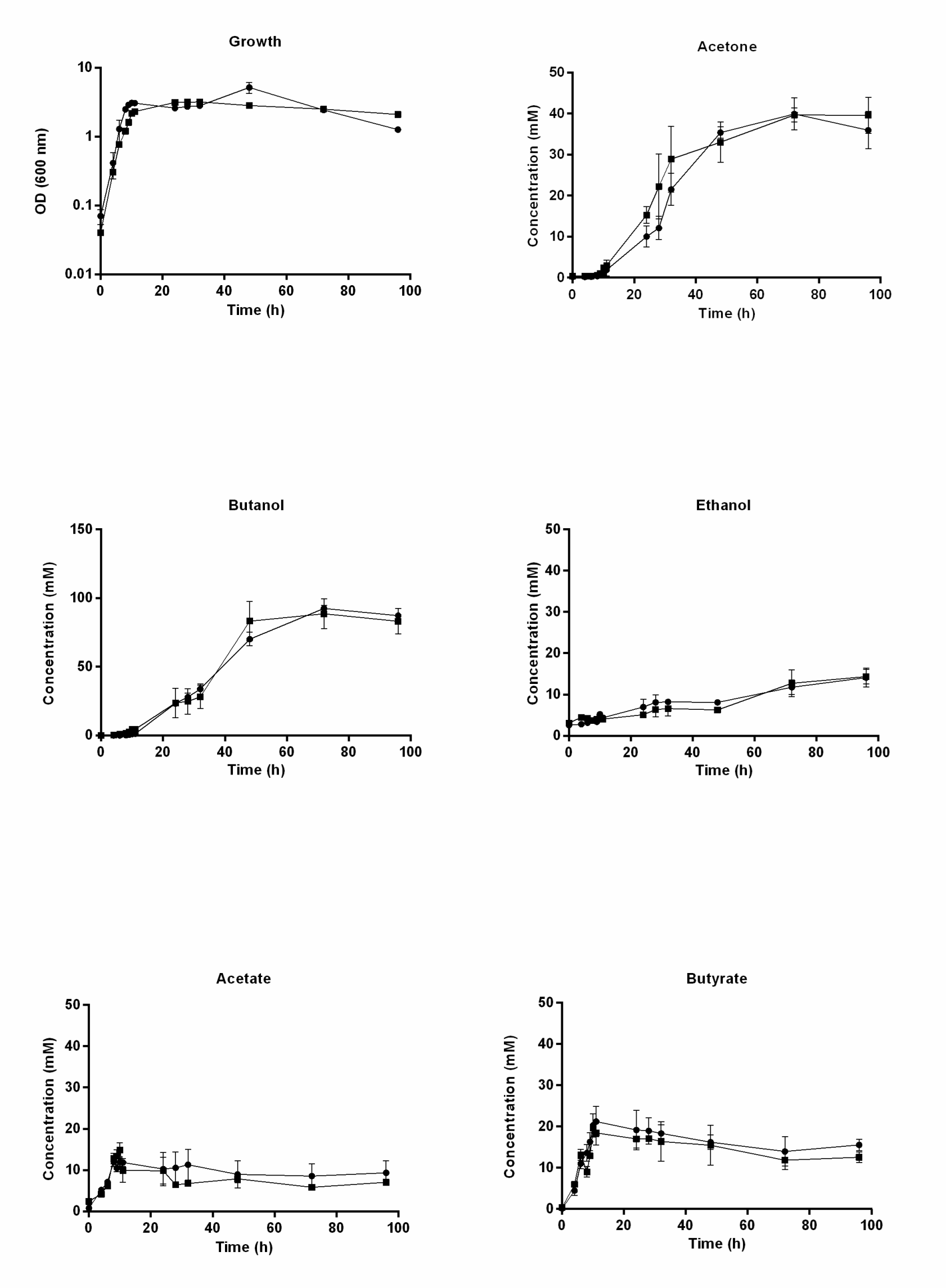

Supplement: S2 Fig — acetobutylicum wild type (black circles) and CRG3011 (black squares). (TIF) [file pone.0122411.s002.tif]

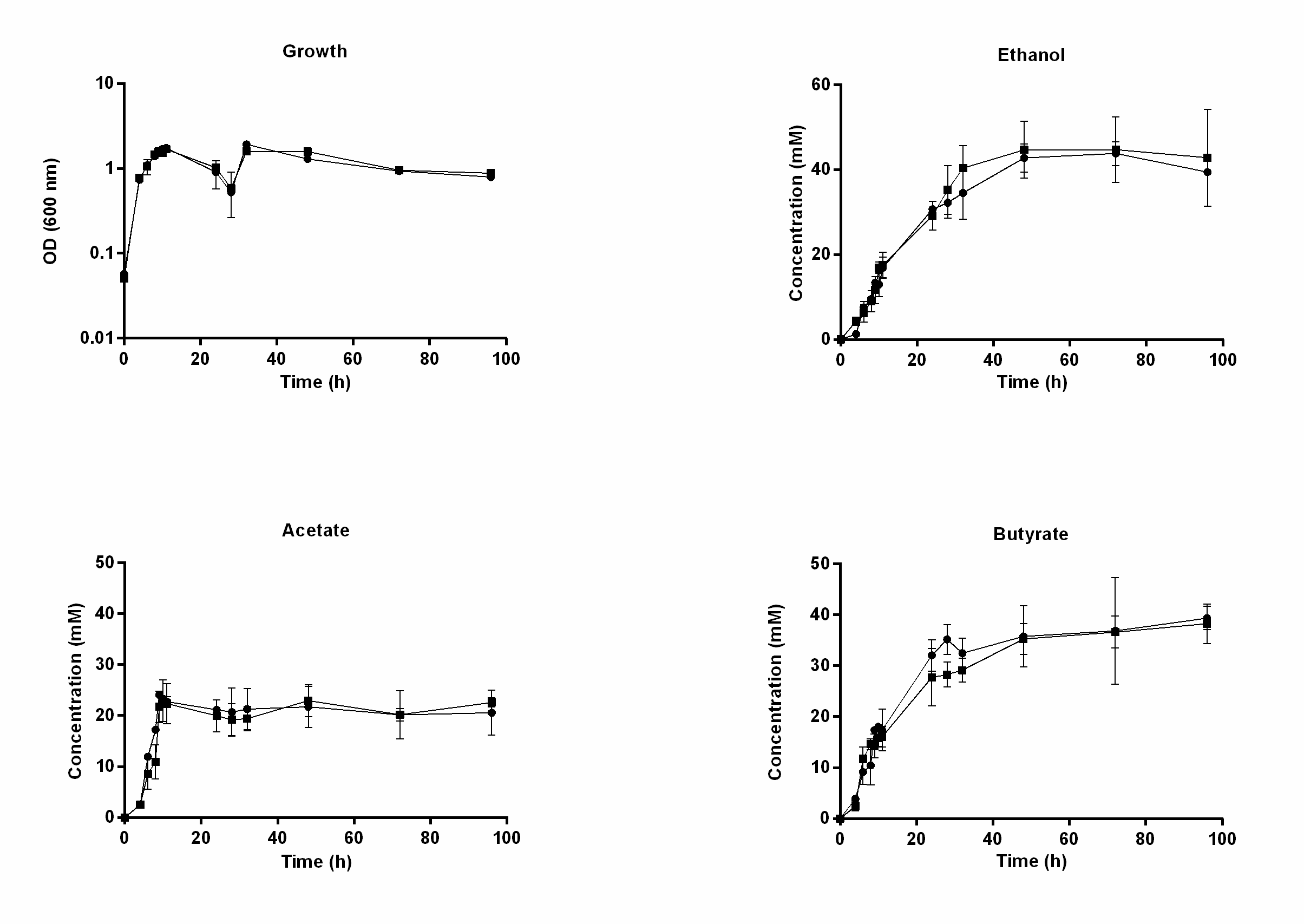

Supplement: S3 Fig — sporogenes wild type (black circles) and CRG3817 (black squares). (TIF) [file pone.0122411.s003.tif]

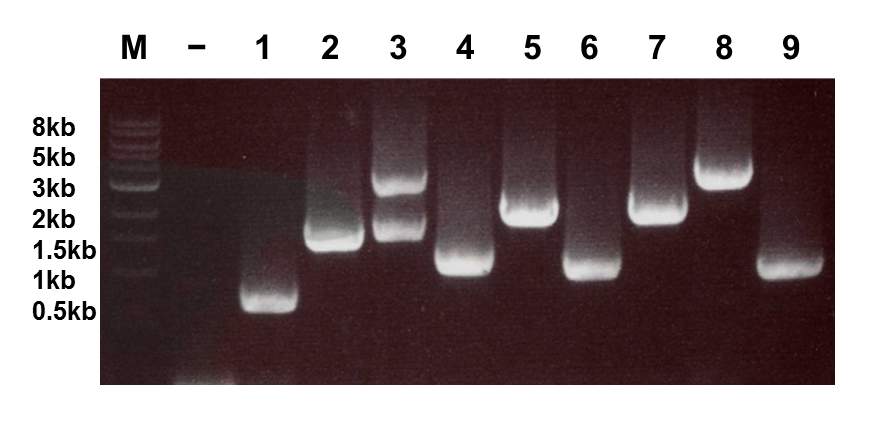

Supplement: S4 Fig — Genomic DNA prepared from each clone was screened for the transposon based insertion. Lane M, 1kb ladder (NEB); lane-, negative control (genomic DNA of CRG3011); lane 1–9, pMTL-YZ14 derived Tm R and Em S clones 1 to 9, clone 3 shows double transposon insertion while the other clones have single insertion. (TIF) [file pone.0122411.s004.tif]
